# Digitalis in Puerperal Fever

**Published:** 1861-02

**Authors:** 


### Digitalis in Puerperal Fever.

This well known arterial sedative is being substituted by some for veratrum in the treatment of inflammatory diseases, with favorable effects.

Dr. Alex. Hadden, House Physician to *Bellevue Hospital*, reports to the *American Medical Times*, "Two cases of puerperal fever successfully treated with digitalis"—Dr. Hadden says:

"The infusion digitalis was substituted for the veratrum veride because of the certainty of its action in my hands in cases of a different character, and without the unpleasant consequences that attend the administration of veratrum viride to the same extent.

"We aimed to reduce the pulse no lower than 60, nor permitted it to rise above 80, without endeavoring to prevent it. Morphia was given with a view to quiet pain effectually. Sulph. quinia was given when the surface of the body was cool and moist, pulse within the above range, even if under the influence of a sedative. Dr. Barker considers the quinia given in large doses, under the above circumstances, in puerperal fever, as not only tonic, but sedative in its effect. I have verified the observation in many cases treated under my charge, and have, moreover, observed that the effects are more lasting."

